# Ataxia Telangiectasia Mutated Protein Kinase: A Potential Master Puppeteer of Oxidative Stress-Induced Metabolic Recycling

**DOI:** 10.1155/2021/8850708

**Published:** 2021-04-01

**Authors:** Marguerite Blignaut, Sarah Harries, Amanda Lochner, Barbara Huisamen

**Affiliations:** Centre for Cardio-Metabolic Research in Africa (CARMA), Division of Medical Physiology, Department of Biomedical Sciences, Faculty of Medicine and Health Sciences, Stellenbosch University, South Africa

## Abstract

Ataxia Telangiectasia Mutated protein kinase (ATM) has recently come to the fore as a regulatory protein fulfilling many roles in the fine balancing act of metabolic homeostasis. Best known for its role as a transducer of DNA damage repair, the activity of ATM in the cytosol is enjoying increasing attention, where it plays a central role in general cellular recycling (macroautophagy) as well as the targeted clearance (selective autophagy) of damaged mitochondria and peroxisomes in response to oxidative stress, independently of the DNA damage response. The importance of ATM activation by oxidative stress has also recently been highlighted in the clearance of protein aggregates, where the expression of a functional ATM construct that cannot be activated by oxidative stress resulted in widespread accumulation of protein aggregates. This review will discuss the role of ATM in general autophagy, mitophagy, and pexophagy as well as aggrephagy and crosstalk between oxidative stress as an activator of ATM and its potential role as a master regulator of these processes.

## 1. Introduction

Ataxia Telangiectasia Mutated protein kinase (ATM) derives its name from the severe, recessive autosomal disease Ataxia-Telangiectasia (A-T). Although this neurodegenerative disease was initially identified in 1926 [1] and described as a clinical entity in 1958 [2], the gene and protein responsible for the disease were only characterized in the early 90's [3–5]. Null mutations in the *Atm* gene that cause the loss of functional ATM, a 370 kDa protein, results in severe characteristic cerebral ataxia and dilated blood vessels present in the conjunctivae of the eyes, also known as telangiectasia [6]. Moreover, nonfunctional ATM has been associated with an increased risk for cancer, radiation sensitivity, endocrine disruption, progressive neurodegeneration, premature ageing, and chromosomal instability (most recently reviewed by Shiloh [7]). The degree of disease severity is dependent on the type of mutation in the *Atm* gene (single or bi-allelic) and heterozygous patients, which make up as much as 1.4-2% of the general population, also exhibit a high incidence of ischaemic heart disease and insulin resistance [8, 9].

Constant oxidative stress is a common denominator in many of the A-T clinical and cellular phenotypes [10]. The loss of functional ATM results in prolonged activation of stress response pathways in the cerebellum but not in the cerebrum or liver [11]. More importantly, this suggests a cytoplasmic role for ATM. The protein resides predominantly in the nucleus of dividing cells [12], where it acts as a transducer in the DNA damage response pathway (DDR), but ATM is mainly found in the cytoplasm of nondividing neuronal cells where it maintains basal metabolic flux [13]. In these cell types, ATM maintains autophagy, a catabolic process that delivers cytoplasmic components for degradation to the lysosome, as well as redox homeostasis, rather than genomic stability and apoptosis. Moreover, it has been suggested that these divergent pathways could be a result of ATM's subcellular localization, as well as different mechanisms of activation and cell survival outcomes [13]. The seminal study of Guo et al. [14] demonstrated for the first time that ATM can be activated in the cytosol in response to exogenous hydrogen peroxide (H₂O₂) independently of DNA damage response, through the formation of a reversible disulphide bond at the only cysteine site within the protein kinase domain, Cys^2991^. Low levels of ROS are sufficient to activate ATM at this residue, independently of the DNA damage response pathway [15], and these distinct activation mechanisms allow ATM to respond to different stresses as well as control different cytoplasmic pathways [16]. More recently, studies showed that ATM can be activated by endogenous ROS including peroxisomal reactive oxygen species (ROS) induced by clofibrate treatment [17] and mitochondrial superoxide induced by low doses of the redox-cycling chemical, menadione [18]. Both peroxisomal and mitochondrial ROS activation of ATM increase autophagy through the activation of AMPK that results in mTOR suppression in the cytosol [19]. Taken together, this suggests that ATM can directly modulate the rate of autophagy in a ROS dependent manner [20] and will be discussed in further detail.

ATM acts as an important sensor of oxidative stress in cells and regulates defences against redox stress [14] by rerouting of glycolysis to the pentose phosphate pathway (PPP) [21] (reviewed more extensively by Blignaut [22]). ATM also regulates mitochondrial biogenesis and DNA content [23] and can lead to mitochondrial dysfunction when absent [24, 25]. Antioxidative treatment that targets the mitochondria in the absence of ATM can decrease the metabolic syndrome, which supports the notion that A-T might be a mitochondrial disease [26, 27]. Importantly, ATM also contributes to glucose homeostasis [28] and is required for the phosphorylation of the insulin-dependent protein kinase, Akt [29, 30].

This review will focus on crosstalk between ROS as an activator of ATM and autophagy as a regulatory mechanism of protein aggregation and oxidative stress in the context of nondividing cells.

## 2. ATM and Oxidative Stress

ATM is a relatively large protein of 370 kDA, consists of approximately 3056 residues and is part of the PI-3 kinase-like protein kinase (PIKKs) family [31]. The catalytic function of ATM identifies with the mechanisms mostly found in serine-threonine proteins that phosphorylate downstream proteins on the hydroxyl group of the serine or threonine residues [32].

The most common function of ATM is to respond to double strand DNA breaks in the nucleus, where the protein is autophosphorylated at Ser^1996^, followed by monomerization of the dimer, and activated in response to DNA damage [33–35]. Upon activation, ATM is responsible for the phosphorylation and activation of downstream proteins, including the Mre11, Rad50, and Nbs1 complex (MRN complex), which aid in DNA repair [35].

Alternatively, ATM can be activated in response to oxidative stress and hypoxic conditions [36, 37], but the question remained whether this can be achieved independently of the DDR pathway. This was answered in a groundbreaking study that reported the direct activation of ATM by hydrogen peroxide (H_2_O_2_) as an inducer of oxidative stress [14]. This study investigated ATM activation under oxidative stress conditions generated with H_2_O_2_ and double strand DNA breaks (DSBs) with bleomycin, a well-known genotoxic agent, in human fibroblasts. Although p53 was phosphorylated at Ser^15^ and Thr^68^ in response to H_2_O_2_ and bleomycin, in an ATM-specific manner, the histone variant, H2AX, as a marker of DNA repair, was only phosphorylated in response to the latter treatment. Inhibition of ATM ablated the phosphorylation of the DNA damage-specific proteins p53, ATM, and Chk2 in the presence of H_2_O_2_, whilst activation of ATM by H_2_O_2_ was inhibited in the presence of the strong hydroxyl scavenger, N-acetylcysteine (NAC). They reported that oxidation resulted in a conformational change in ATM but not the monomerization observed in response to DNA damage. The study found that ATM forms a reversible disulphide bond at the cysteine site, Cys^2991^, and mutation of this site from Cys^2991^ to Ala^2991^, resulted in a construct that can be activated in the presence of DSBs but not oxidative stress. Although ATM contains several disulphide bonds, it is the covalent disulphide bond at Cys^2991^ through which ROS modulates its effects.

However, it should be noted that the interplay between oxidized ATM and DSB-activated ATM is complicated: Guo et al. [14] suggested that oxidative stress disrupts DNA binding at the complex responsible for ATM recruitment to the damaged site and can therefore inhibit ATM activation by DSBs, resulting in the oxidation of ATM under high ROS conditions. A more recent study showed that excess endogenous ROS represses ATM-dependent homologous DNA repair in cells obtained from ataxia patients with oculomotor apraxia type 3 (AOA3 cells) which has implications for both neurodegeneration and tumorigenesis [38]. Irrespective of the lack of consensus with regard to the oxidation of ATM under either high or low ROS conditions, many of the ATM substrates identified with proteomic analyses, implicate ATM in metabolic signalling pathways [39].

Under normal physiological conditions, ROS act as signalling intermediates in many cellular processes to induce redox homeostasis. On the other hand, elevated ROS levels, aptly described as oxidative stress, have been linked with over 150 diseases, most notably atherosclerosis, diabetes, and cancer [19]. It has therefore been suggested that A-T might, in essence, be an oxidative stress disorder [40]. In order to understand how ATM contributes towards the maintenance of basal metabolic flux and redox homeostasis, a short overview of oxidants and their cellular targets is required.

Briefly, ROS derive from the reduction of molecular oxygen which most notably includes oxygen (O_2_^•-^), hydroxyl (^•^OH), peroxyl (RO_2_•), and alkoxyl (RO•), as well as certain nonradicals that are either oxidizing agents or can be converted into radicals such as hypochlorous acid (HOCl), ozone (O_3_), single oxygen (ᶦO_2_), and H_2_O_2_ [41]. Metabolism of nitric oxide (NO) results in the formation of reactive nitrogen species (RNS) that can either contribute to oxidation, nitrosation, or nitration [42]. The enzymatic action of nitric oxide synthase (NOS) results in the formation of nitric oxide (NO) but can also produce O_2_^•-^ under the right circumstances. A rapid reaction between NO and O_2_^•-^ results in the formation of peroxynitrite (ONOOH) which is involved in oxidation, nitrosation, and nitration. In the case of nitration, nitrotyrosine can be formed and alter cell signalling pathways. For example, nitrite together with HOCl has been detected in diseased human vascular tissue and drives the formation of artherogenic LDL which is implicated in atherosclerosis [43].

There are numerous sources of endogenous ROS including the cytoplasm, where O_2_^•-^, generated by either mitochondria or the NOX-family (nicotinamide adenine dinucleotide phosphate (NADPH) oxidases), is converted to H_2_O_2_, as well as the production of H_2_O_2_ by the endoplasmic reticulum (ER) as a byproduct of protein oxidation and as an end product in several peroxisomal oxidation pathways including *β*-oxidation of long-chain fatty acids [44, 45]. NOX1, -2, -4, and -5 transport electrons across biological membranes in order to reduce oxygen to superoxide and are expressed throughout the cardiovascular system, brain, and cerebrovascular tissue (extensively reviewed by [41]). This protein family is one of the best known sources of cytoplasmic ROS, which in itself has been described as the cornerstone of cellular signalling and disease pathophysiology [46–48]. The broad impact of ROS is made possible by the large number of molecules that ROS can interact with, including small organic molecules, proteins, lipids, carbohydrates, and nucleic acids. These interactions can either destroy or irreversibly change the function of the target molecule and accordingly contribute towards pathogenesis [41].

Most redox reactions, however, occur through the reversible reduction and oxidation of crucial reactive cysteine residues that form thiolate anions at a physiological pH [49]. Oxidation of this residue, as is the case for ATM at Cys^2991^, results in a sulfenic residue (SOH), which is further modified to form an intramolecular disulphide bond. As mentioned previously, the addition of exogenous H_2_O_2_*in vitro* forms an active ATM dimer of two covalently-linked monomers. Possible *in vivo* sources of oxidants, that can reduce thiol and oxidizing disulphide bonds, are generated by the membrane bound NOX-family of NADPH oxidases. These enzymes produce anions that can be dismutated into H_2_O_2_ which selectively re-enters the cell through aquaporin channels [44].

NOX-4, which is located in close proximity of the nucleus in a wide range of human cells, produces ROS innately and is elevated in A-T cells [50]. Specific inhibition of both NOX-4 and NOX-2 alleviates increased cancer risk in A-T null mice, whilst the inhibition of ATM increased NOX-4 expression in normal cells. NOX-4 is thus potentially a critical mediator of ROS and in the development of A-T.

Most recently, Zhang et al. [18] reported that ATM acts as a redox-sensor in response to endogenous mitochondrial ROS (H_2_O_2_) and serves as a critical juncture in the regulation of carbohydrate metabolism. The study showed that glutathione production, which is also an endogenous antioxidant, is increased in cells expressing an ATM Cys^2291^Ala mutant construct and suggests that this is an attempt to compensate for a lower glucose flux through the PPP, thus decreasing the availability of NADPH.

Taken together, ATM can be activated in response to exogenous (H_2_O_2_) and endogenous ROS (mitochondrial) as well as through NADPH-oxidases, allowing it to respond as a redox-sensor for the PPP [16]. However, ATM has also been shown to mediate autophagy in response to oxidative stress, which will be discussed in the following section.

## 3. ATM-Mediated Autophagy

Autophagy is a highly regulated catabolic process that literally translates to “self-eating”; this general term describes the delivery of cytoplasmic components, including parts of the cytosol and large protein complexes, within a double membrane vesicle (autophagosome) to the lysosome for degradation [51]. Basal physiological autophagy ensures cellular homeostasis and protein recycling within all eukaryotic cells [52] but can also be stimulated in response to cellular stress, including but not limited to, oxidative stress, hypoxia, nutrient starvation, DNA damage, and protein aggregation [53]. The ubiquitin-proteosome system (UPS) targets only individual, short lived, or misfolded proteins for degradation, whilst autophagy recycles larger components such as damaged organelles, excessive, or toxic byproducts and larger protein complexes and aggregates [54]. This diverse but specific degradation response is enabled by three types of autophagy, namely, macroautophagy, microautophagy, and chaperone-mediated autophagy, which differ with regard to their targeted substrate and sequestration mechanism [55]. This review will focus on the role of ATM in ROS-induced macroautophagy.

The initiation of macroautophagy occurs in the cytoplasm via the activation of adenosine 5′-monophosphate- (AMP-) activated protein kinase (AMPK) in response to nutrient starvation and hypoxia [56]. Moreover, AMPK activation results in the inhibition of lipid and glycogen synthesis, whilst concurrently activating free fatty acid oxidation and glycolysis [56]. Moreover, the activation of AMPK phosphorylates and activates TSC2 (tuberous sclerosis complex 2) resulting in the repression of mTOR complex 1 (mechanistic target of rapamycin complex 1), which is a negative regulator of autophagy [57]. AMPK activation can also phosphorylate the mTOR-binding partner, raptor, and induces 14-3-3 binding to raptor, which is required for the inhibition of mTORC1 [58], as well as phosphorylate mTORC1 directly at Thr^2446^ [59]. Once mTORC1 is repressed, unc-51-like kinase (ULK1) is dephosphorylated and consequently activated. Under starvation conditions, AMPK promotes autophagy through the direct phosphorylation of ULK1 at Ser^317^ and Ser^777^, whereas sufficient nutrients promote mTOR activity and prevents ULK1 activation through phosphorylation at Ser^757^, consequently disrupting the interaction between ULK1 and AMPK [60]. ULK1, together with Atg1, form one of at least five core molecular components that is required for the formation of the autophagosome membrane [61]. The other core molecular components include the Beclin1/class III PI3K complex; the transmembrane proteins, Atg9 and vacuole membrane protein 1 (VMP1); and two ubiquitin-like protein conjugation systems, Atg12 and Atg8/LC3 [62]. Once the formation of a phagophore is initiated, the vesicle expands sequentially and engulfs the cytosolic cargo, in either a selective or nonselective manner, to form the autophagosome [63]. The formation of the autophagosome is driven by the Atg (AuTophagy related) proteins and has been reviewed extensively [63, 64].

Selectivity of the targeted cargo is conferred by receptors that recognize and interact with lipidated ATG8 family proteins, which are located on the concave side of the developing autophagosome [65]. This interaction is enabled by LC3 interacting regions (LIR) that bind to the LIR docking sites of ATG8 family proteins [65]. The ATG8 family consists of the LC3/GABARAP protein family and includes the microtubule-associated protein 1 light chain 3 (MAP1LC3A-B and C) or *γ*-aminobutyric acid (GABA) type A receptor–associated protein (GABARAP and GABARAP-like 1 and -2) [66]. Of these proteins, the best-studied protein is LC3B, which confers selectivity, together with the GABARAP proteins, for pexophagy and mitophagy through interaction with adapter proteins [66]. LC3B associates with the forming autophagosomal membrane through the formation of a covalent bond to phosphatidylethanol (PE) enabled by a ubiquitination-like sequence of enzymatic events where ATG7 acts as the LC3 activating enzyme and ATG 3 as the conjugating enzyme that transfer LC3 to PE to form lipidated LC3-PE/LC3-II [67, 68].

Autophagy adapter proteins interact directly with the ATG8 proteins and share the ability to interact simultaneously with the autophagosome through interaction of their LIR motif with LC3 as well as the cargo substrate, which is often ubiquitylated [67]. These receptors include, amongst others, p62/SQSTM1, BNIP3 (BCL2/adenovirus E1B 19 kDa interacting protein), FUNDC1 (Fun14 domain containing 1), NBR1 (neighbour of BRCA1), NDP52 (nuclear dot protein of 52 kDa), and optineuron, of which many have a ubiquitin-binding domain (UBD) that can interact with different ubiquitin chain linkages associated with the targeted cargo and in doing so, provide selectivity [67]. Once the cargo is tethered to the forming autophagosome, LC3B and the GABARAP subfamily promote the elongation and fusion (closure) of the autophagosome [66], which can then fuse with the lysosome to form an autolysosome and results in pH changes to occur in the lumen of the lysosome [69]. The change in lysosomal pH is essential for successful protein degradation as the hydrolyses responsible for cargo breakdown are activated in an acidic environment [70]. The process of autophagosome maturation, trafficking, and lysosomal fusion as well as the proteins involved in this process has recently been reviewed extensively [71].

In view of the oxidative stress induced activation of ATM, as well as the pathophysiology associated with elevated ROS in ATM-deficient cells, Alexander et al. [19] reported that the activation of oxidized ATM increases autophagy through the activation of TSC2 via the liver kinase B1 (LKB1)/AMPK pathway, resulting in the repression of mTORC1. Moreover, inhibition of mTORC1 with rapamycin results in the concomitant improvement of ROS levels in ATM^−/−^ mice. The authors found that low concentrations of H_2_O_2_ rapidly induced mTORC1 repression that could, in turn, be rescued by the addition of NAC or pretreatment with catalase. Of relevance as well, is that chemical mitochondrial uncoupling which depletes the antioxidant, glutathione, also repressed mTORC1 signalling, indicating that both exogenous and endogenous ROS activation of ATM can induce mTORC1 repression.

The same research group found that nitrosative stress (nitric oxide (NO)) also activates ATM and results in the phosphorylation of AMPK through LKB1, activation of the TSC2 complex and consequent repression of mTORC1 [72]. ATM-mediated repression of mTORC1 decreased phosphorylation of direct target proteins of mTORC1 such as 4E-BP1 (4E-binding protein 1), S6K (ribosomal S6 kinase), and ULK1 (Unc-51 like autophagy activating kinase). Consequently, nitrosative stress-mediated activation of ATM can increase autophagy by decreasing mTORC1 mediated phosphorylation of ULK1 at Ser^757^ and increasing ULK1-phosphorylation at Ser^317^ by AMPK. However, the precise mechanism through which NO activates ATM is still unknown. Induction of autophagy by NO also resulted in decreased cell viability, which suggests a cytotoxic response.

LKB1 can be phosphorylated directly at Thr^366^ by active ATM in response to ionising radiation (IR) [73] as well as through oxidative stress as discussed above, consequently activating AMPK directly to modulate apoptosis [74] or autophagy in the event of energetic stress.

On the other hand, one of the key roles of AMPK in cardiac tissue is the response to hypoxia/ischaemia, which is also under the direct control of LKB1; in the absence of LKB1, mouse hearts show increased mTORC1 signalling and protein synthesis that can lead to hypertrophy [75]. Interestingly, Emerling et al. [76] showed that the hypoxic activation of AMPK in mouse fibroblasts is dependent on mitochondrial oxidative stress that is generated by the ETC and not the cytosolic adenosine monophosphate (AMP)/adenosine triphosphate (ATP) ratio. Although they did not evaluate the role of ATM in their study, it supports the notion that the oxidative activation of ATM, due to increased mitochondrial dysfunction, can potentially mediate the activation of AMPK in response to hypoxic stress.

In a nutshell, autophagy is a catabolic process responsible for the degradation and recycling of damaged organelles and is central to the maintenance of cellular homeostasis. The activation of ATM through ROS and NO places ATM directly upstream of AMPK, which in turn, drives the inhibition of mTORC1 and upregulation of autophagy through ULK1. This allows cells to eliminate damaged organelles that can drive increased oxidative stress and recycle these components to maintain nutrient and energy homeostasis, but this process can also be mediated independently of ATM. ROS-induced autophagy can be induced by either O_2_^•-^ [77] or H_2_O_2_ [78] that is produced in response to either glucose or nutrient starvation and can cause mitochondrial energetic stress due to decreased ATP availability [52]. Both redox balance and ROS formation can be regulated by changes in the autophagy rate and consequently either directly regulate mitochondrial homeostasis or indirectly regulate mitochondrial function [79].

Key to effective autophagy, which is also responsible for the degradation of ATM [80], is the fusion of the autophagosome to lysosomes, in order to form an autolysosome where the targeted content is degraded. Recent observations in ATM^−/−^ neurons showed upregulated autophagic flux of lysosomes with a more acidic pH and led to the finding that the ATPase, H^+^ transporting lysosomal V1 subunit A (ATP6V1A proton pump) is a target of ATM [80]. The absence of ATM results in the peri-nuclear accumulation of lysosomes which suggests that this could be due to a physical interaction between ATM and the retrograde transport motor protein, dynein. Lysosomal dynein accumulates in ATM ^−/−^ mouse brains indicating that ATM inhibits axonal transport through dynein motor proteins. Similarly, the study found that the loss of ATM resulted in the impaired glucose uptake due to the inhibition of the translocation of the SLC2A4/GLUT4 (solute carrier family 4 (facilitated glucose transporter) 4) to the plasma membrane, and increased trafficking to lysosomes instead [80]. This observation further supports previous reports that decreased ATM activity is associated with metabolic syndrome [81] and insulin resistance [82]. The importance of ATM in autophagy is highlighted by the accumulation of lysosomes, as well as increased oxidative stress in the cerebellum of ATM-null mice [83].

Increased oxidative stress and a weakened antioxidant defence due to dysfunctional autophagy can induce cellular damage and result in neuronal cell death, which is the major causative factor in the development of Parkinson's disease, which predominantly affects aged individuals above 60 [71]. Increasing age results in a decline in autophagy as well as an increase in protein misfolding and oxidative stress [67] and can lead to the disruption of cellular homeostasis [68]. Similarly, age-associated decreases in ATM protein levels [84] may result in the development of metabolic syndrome, lysosomal accumulation, and protein aggregation that are associated with age-related neuronal diseases [85] and the development of cardiac dysfunction including fibrosis and hypertrophy [86].

## 4. ATM and Aggrephagy

Protein aggregates develop when proteins are misfolded due to mutations, incomplete translation, inappropriate protein modifications, oxidative stress, and ineffective assembly of protein complexes [87]. The accumulation of misfolded and dysfunctional protein aggregates, often due to oxidative stress [88, 89] or downregulated or disrupted autophagy [90], can be toxic to the cell and cause a disruption of cellular homeostasis that is detrimental to cellular survival in many diseases, and in particular, neurodegenerative diseases [91]. Aggregation is driven by exposed hydrophobic patches in misfolded proteins that sequester other proteins. Misfolding can be repaired by molecular chaperones, but if the damage is too great, the misfolded proteins are guided by chaperone complexes for degradation by either the ubiquitin-proteosome system (UPS) or the lysosome through chaperone mediated autophagy or aggrephagy [87]. The latter process specifically refers to the selective sequestration of protein aggregates by macroautophagy and will be the discussed further.

Protein aggregation is classically associated with neurodegeneration but has been observed in nearly every cardiometabolic disease [92]. The accumulation of proteins and dysfunctional organelles contributes to the development of pathology in almost all tissues and thus requires a very fine balance between apoptosis and autophagy [93]. There seems to be synergistic roles for ATM and p53 with regard to the regulation of autophagy, where ATM regulates mitochondrial homeostasis and oxidative stress in order to prevent cells from undergoing apoptosis in response to nongenotoxic p53 activation [94]. Genetic or pharmacological loss of ATM kinase activity blocks autophagy and increases ROS, which is sufficient to commit cells to apoptosis in response to Nutlin 3 treatment, an inhibitor of the p53 E3 ubiquitin ligase MDM2 that activates p53 [94].

Most recently, it has been shown that the loss of function mutation that blocks ATM activation by oxidative stress, but not genotoxic stress, results in widespread protein aggregation, especially when cells are exposed to low levels of ROS, and includes polypeptides mainly implicated in DNA metabolism and gene expression [95]. This implicates a role for ATM in protein homeostasis. Moreover, protein aggregation is very relevant to neurodegeneration, especially with regard to the loss of function of Purkinje neuronal cells, which is a hallmark of A-T [96].

Proteasomal degradation is also required for the maintenance of autophagy at physiological levels as is the case with ULK1; it is specifically ubiquitinated by the E3 ligase NEDD44 that marks it for proteasomal degradation, whilst still being actively translated and transcribed [75]. The transcription of ULK1 is in turn inhibited by mTOR during prolonged autophagy and allows for the maintenance of ULK1 protein at basal levels within the cell.

It is also possible that ATM can play a more active role in ULK1 phosphorylation through p32. Although p32 was first recognized as a novel substrate of ATM in cardiac DNA damage [97], it has recently been identified as a regulator of ULK1 stability [98]. In a study that investigated the cardiotoxicity and genotoxic effect of chemotherapeutic agents that induce cell death through the ATM-mediated phosphorylation of p53, the protein, p32 (CIQBP/HABP1), was identified as an endogenous substrate in mouse hearts [97]. The protein is phosphorylated at Ser^148^ by ATM in response to genotoxic stress, but the authors did not comment on the physiological effect thereof [97]. However, p32 has been found to be essential for maintaining the activity and stability of ULK1 [98]. The study found that the ablation of p32 results in increased proteolysis of ULK1, that consequently impaired starvation-induced autophagic flux as well as the clearance of damaged (uncoupled) mitochondria, and highlights the importance of p32 for ULK1 activity. The phosphorylation of ULK1 by AMPK also regulates the translocation of ULK1 to mitochondria in response to hypoxia [99] where it phosphorylates the autophagy cargo receptor, FUNDC1 [100], and regulates mitophagy [101]. Although ATM was not investigated in this context, it is tempting to hypothesize that ATM could influence ULK1 potentially through the phosphorylation of p32 in the heart.

## 5. ATM Mediates Selective Autophagy

Constitutive autophagy plays a protective role in mitochondrial rich cardiomyocytes, where accumulation of abnormal proteins and organelles, especially mitochondria, may directly cause cardiac dysfunction [102]. Mitophagy, a specialized mechanism of autophagy that specifically aims to degrade and maintain mitochondrial quality, is central to maintaining cellular integrity and cellular homeostasis [103]. The process of mitophagy is known to decrease during ageing, thus resulting in mitochondrial dysfunction [104].

Classically, depolarized mitochondria initiate the accumulation of (PTEN-) induced kinase 1 (PINK1) on the outer mitochondrial membrane [105]. PINK1 is degraded in healthy mitochondria but accumulate on the outer mitochondrial membrane of damaged mitochondria that, in turn, drives the recruitment and translocation of Parkin [85] to the mitochondria. Parkin is an E3 ubiquitin ligase which is phosphorylated by PINK1, stimulating its translocation to the mitochondria where it ubiquinates several outer mitochondrial membrane proteins. This promotes further PINK1 phosphorylation and the formation of ubiquitin chains that localizes mitophagy receptors that contain UBDs to Parkin-ubiquitylated mitochondria, including p62 (SQSTM1), NBR1, and optineurin [106] that can attach to autophagosomal membranes and envelop the damaged mitochondria (reviewed by Nguyen et al. [107]) for degradation [108].

PINK1 also phosphorylates the fusion protein, mitofusin 2 (Mfn2), which can serve as a mitochondrial receptor for Parkin, promoting its ubiquitination [88]. The loss of Mfn2 prevents the translocation of Parkin in depolarized mitochondria and suppresses mitophagy, which drives the accumulation of dysfunctional mitochondria and decreased mitochondrial respiration in mouse cardiomyocytes [88]. Alternatively, mitophagy can be mediated by mitophagy receptors. Mitochondrial receptor-mediated autophagy is mediated by the pro-apoptotic proteins, BNIP3 and NIX, that localize to the outer mitochondrial membrane and act as receptors for targeting autophagosomes through direct interaction of conserved LC3-interacting regions (LIRs) with LC3 on the autophagosome, often in response to hypoxia [109] and in the absence of mitochondrial membrane permeabilization [110]. FUNDCI is an outer mitochondrial membrane protein that has been implicated in hypoxia-mediated mitophagy in mammalian cells [111]. Similar to BNIP3 and NIX, FUNDC1 acts as a receptor for the autophagosomal membrane and interacts directly with LC3 through LIR. The serine/threonine protein phosphatase, PGAM5, dephosphorylates FUNDC1 during hypoxia or mitochondrial membrane depolarization and promotes interaction with LC3 with consequent mitophagy [112].

**Figure 1:**
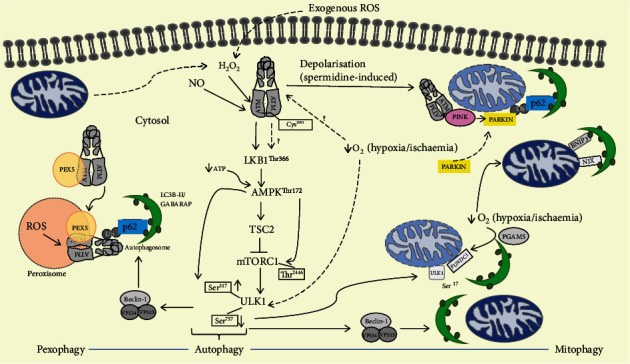
ROS can activate cytosolic ATM. ATM is activated in response to both endogenous and exogenous ROS, as well as NO at Cys^2991^, where it forms a disulphide bond. Once activated, it phosphorylates LKB1 at Thr^366^ which phosphorylates AMPK and drives the inhibition of mTORC1 through TSC2. The inhibition of mTORC1 phosphorylates ULK1 at Ser^757^, whilst AMPK phosphorylates ULK1 at Ser^313^. This initiates autophagy and the formation of an autophagosome that targets peroxisomes specifically for degradation through the ATM-mediated ubiquitination of PEX5. It is currently unknown whether ATM is involved in either the activation of AMPK or suppression of mTOR in response to ROS to induce mitophagy. ATM mediates PINK/Parkin mitophagy pathway in response to spermidine treatment, which induces ROS and consequently activate ATM, that is then recruited to the permeabilized mitochondrial membrane where it colocalize with PINK and drives the recruitment of Parkin which is ubiquitinated. The ubiquitin chain binds to LC3 (green balls) on the autophagosome, which then engulfs damaged mitochondria for lysosomal degradation (not shown). Hypoxia or mitochondrial uncoupling can also activate ULK1, driving its translocation to the damaged mitochondrion membrane where it phosphorylates FUNDC1, which enhances its binding to LC3, whereas the dephosphorylation of FUNDC1 by PGAM5 also allows FUNDC1 to directly interact with LC3. BNIP and NIX can act as mitochondrial receptors in response to hypoxia when the mitochondrial membrane is not permeabilized and bind to LC3 on the autophagosome. Damaged mitochondria produce less ATP that activates AMPK, which in turn phosphorylates ULK1 and activates the Beclin1-VSP34-VSP15 complex and drives the formation of an autophagosome. Damaged mitochondria can also produce ROS which inhibits mTOR and leads to the activation of autophagy.

More recently, a direct link between ATM and PINK1/Parkin recruitment was shown, as ATM was able to initiate the accumulation of PINK1 and translocation of Parkin in the presence of spermidine and lead (Pb) initiating mitophagy [113, 114]. Spermidine is a natural polyamine involved in several biological processes including cell proliferation and apoptosis and tends to decline with age [115]. Spermidine also elicits mitochondrial depolarization that causes the formation of mitophagosomes and mitochondrial targeted lysosomes, which has been suggested to occur via ATM-dependent activation of the PINK1/Parkin mitophagy pathway [114]. Spermidine-induced mitochondrial depolarization is abrogated in the presence of the chemical ATM inhibitor, KU55933. Moreover, spermidine promotes the colocalization of phosphorylated ATM and PINK1 on the outer mitochondrial membrane, which, together with the translocation of Parkin, can be blocked by the ATM inhibitor. The authors suggest a model whereby activated ATM drives PINK1 accumulation as well as Parkin translocation with consequent mitophagy in response to spermidine treatment (Figure 1).

ATM may therefore be central to mitophagy by directly activating the pathway or by indirectly activating autophagy in response to oxidative stress. Thus, if pathological ATM signalling occurs, mitophagy could be affected, predisposing the cell to mitochondrial oxidative stress [22]. ATM is also activated by nitrosative stress and contribute to sustained mitophagy of damaged mitochondria through the newly characterized ATM-denitrosylase S-nitrosoglutathione reductase (GSNOR) axis [116].

The chronic oxidative stress observed in A-T has led to the suggestion that A-T might be a mitochondrial disease [40] and has also been linked with intrinsic mitochondrial dysfunction [24]. The latter study found that lymphoblastoid cells from A-T patients contain an increased population of mitochondria with a decreased membrane potential, when compared to control cells. Proteins with specific roles in mitochondrial DNA damage and/or ROS scavenging, including mnSOD, peroxiredoxin 3, and mitochondrial topoisomerase, were also elevated in these cells. Indeed, the decreased membrane potential translated into decreased respiratory activity in the A-T cells compared to the wild type controls. Concomitantly, the authors showed that the *in vivo* loss of *ATM* in mice resulted in mitochondrial dysfunction in thymocytes that was accompanied by increased mitochondrial content and mitochondrial ROS due to a decrease in mitophagy. Interestingly, they observed a significant decrease in complex I activity as well as ATP production and an increase in oxygen consumption. The study also found that autophagy was not affected by the absence of ATM and suggested that changes in mitochondrial dynamics such as fission and fusion could contribute towards defective mitophagy. The authors concluded that the observed defects in the absence of ATM suggest that ATM might localize directly to mitochondria. Fractionation studies in cells revealed that the mitochondrial fraction of HepG2 cells was enriched with ATM and activated ATM in response to H_2_O_2_ treatment [117]. In contrast to previous observations that ATM associated with the peroxisomal fraction [118], Morita et al. [117] detected almost no ATM in this fraction. This reverberates with the suggestion [119] that both the cell type and culture conditions of immortalized A-T cells can affect mitochondrial homeostasis and autophagic responses which explain the differences in mitochondrial content reported in A-T deficient cell lines.

Mitochondrial respiration inhibition can also lead to increased mitochondrial ROS production. Treatment of HeLa cells with either rotenone or Antimycin C failed to increase mitochondrial hydrogen peroxide production although it did increase mitochondrial superoxide production [18]. Superoxide itself failed to drive ATM dimerization and suggested that mitochondrial superoxide must be converted to H_2_O_2_ in order to activate ATM in either the cytosol or nucleus of HeLa cells.

Our group reported that ATM is directly associated with the inner mitochondrial membrane of cardiac mitochondria, and the inhibition thereof decreases oxidative phosphorylation and the ATP synthesis rate in a complex I-mediated manner [120]. Similarly, ATM^−/−^ thymocytes exhibit decreased complex I activity [25], whereas the chemical inhibition of ATM resulted in a posttranslational decrease of COX-IV [121]. This is interesting because the inhibition of COX-IV has been associated with increased ROS production at complex I, albeit in the mitochondrial matrix [122]. Depletion of ATP in neuronal Purkinje cells results in increased ROS production that can activate ATM, consequently leading the phosphorylation of Nrf1 that specifically upregulates the expression of nuclear-encoded mitochondrial genes and improves electron transport chain capacity and restores mitochondrial function [123]. Similarly, Fang et al. [124] reported increased mitochondrial content in ATM-knockdown (ATM-KD) rat neurons coupled to increased ROS production. The authors suggested that this could reflect decreased ATP production and either inadequate or inefficient mitophagy. Moreover, the study showed that mitophagy is suppressed in ATM-KD HeLA cells and rat neurons but that the phenotype could be rescued by replenishing cellular NAD^+^ which significantly improved life-span in ATM^−/−^ mice [124].

Interestingly, Beclin-1 heterozygosity in ATM^−/−^ mice reduces mitochondrial ROS and complex I abnormalities in thymocytes [25]. Beclin-1 forms part of the complex required for the induction of autophagy [125] but is also required for the recruitment of Parkin to the mitochondrial membrane where it induces ubiquitination and proteasomal degradation of proteins on the outer mitochondrial membrane [126]. This leads to the inhibition of fusion and the trafficking of dysfunctional mitochondria [126]. It is still unclear why the allelic loss of Beclin1 would promote improvement of mitochondrial dysfunction in ATM^−/−^ mice, but it has led to the suggestion that Beclin-1 might have additional functions besides its role in autophagy [25].

Terminally differentiated cells such as cardiomyocytes and neuronal cells are dependent on the efficient removal and replacement of dysfunctional mitochondria to ensure cell survival and to maintain cellular homeostasis [111, 127]. A decrease in ATP production and increased ROS production as indicators of mitochondrial dysfunction can result in either the release of apoptotic proteins or the selective clearance of the damaged mitochondria. Mitophagy thus serves as an early cardioprotective response through the removal of damaged mitochondria, and if this fails, apoptosis can be induced in response to excessive oxidative stress [128]. Moreover, reduced autophagy, together with the accumulation of dysfunctional mitochondria, has been associated with heart failure and aging [111].

Pexophagy is the targeted selective degradation of peroxisomes [129] and is another example of selective autophagy [130]. Peroxisomes utilise *β*-oxidation to reduce long-chain fatty acids into medium length fatty acids that can be shuttled to the mitochondria. These highly metabolic organelles generate ROS during *β*-oxidation and require homeostatic maintenance to prevent oxidative stress. ATM binds to the peroxisome importer receptor, PEX5, in response to excessive ROS and mediates peroxisome-specific autophagy (pexophagy) by phosphorylating PEX5 at Ser^141^ and promoting mono-ubiquitylation at Lys^209^, whilst simultaneously inducing autophagy through the activation and phosphorylation of TSC2 and ULK1 [17, 131, 132]. Ubiquitylation of PEX5 is mediated by the complex PEX2-PEX10-PEX12 and is then recognized by the autophagy adapter proteins, p62 and NBR1, which directs the autophagosome to the peroxisomes for pexophagy [129].

Loss of function mutations in ATM, such as the ability to sense oxidative stress, can result in a reduction in mitochondrial antioxidant defences, lead to the accumulation of ROS and oxidative damage to mitochondria and other cellular components [18], as well as protein aggregation [95]. Selective autophagy seems to be mainly mediated by ubiquitination which is essential for conferring selectivity [133], as is the case of ATM-mediated pexophagy. As previously discussed, this also implies a potential role for ATM in aggrephagy (degradation of damaged or misfolded proteins) which is dependent on p62 ubiquitination [92].

## 6. Conclusion

This broad overview describes the apical protein, ATM protein kinase, at the nexus of oxidative stress-induced autophagy [14, 18] as well as nitrosative stress-induced autophagy [72, 116], mitophagy [113, 114], and pexophagy [17, 132] mainly in the context of nondividing cells such as cardiomyocytes and neurons. Site-specific mutations that renders ATM insensitive to oxidative stress increase protein aggregation [95], whilst loss of function increases peri-nuclear lysosomal accumulation [80] as well as mitochondrial oxidative stress [25] and dysfunction [24, 25, 120]. Cytoplasmic ATM thus plays a central role in redox homeostasis and ROS-mediated autophagy.

As a master regulator of DNA repair, activation of ATM by exogenous and endogenous oxidative stress, independently of DNA strand breaks, only recently came to light [14, 18]. This finding paved the way to understanding the severe neurodegeneration and associated protein aggregation observed in A-T patients that is largely due to disrupted ATM protein kinase functioning leading to disrupted autophagy, mitophagy, and pexophagy [80, 95]. Additionally, the regulation of ATM levels by autophagy [80] and the role of ATM in oxidative stress-mediated autophagy in an AMPK/mTORC1 dependent manner were discovered [13, 19, 132]. Similarly, ROS-induced pexophagy is modulated by ATM through the TSC2/AMPK/mTORC1 pathway in which the disruption of this signalling pathway leads to interrupted cellular homeostasis causing pathologies linked to neurodegeneration [17, 131, 134].

It has been suggested that the pathogenesis of A-T could be ascribed to excessive ROS and that A-T might therefore be an oxidative stress disease [40]. Several studies have investigated the effect of the absence or inhibition of ATM on mitochondrial function and found that ATM is innately associated with the inner mitochondrial membrane and oxidative phosphorylation of cardiac mitochondria [120]. In addition, the absence of functional ATM in the mitochondria of ATM-null thymocytes and fibroblasts was associated with decreased ATP production, increased ROS production [24, 25], and a decrease in mitophagy [119, 123].

Therefore, activation of ATM by oxidative stress and the consequent maintenance of redox homeostasis through autophagy, pexophagy, aggrephagy, and mitophagy place ATM at the centre of cross-talk between ROS and autophagy signalling.
